# Proton therapy versus photon radiation therapy for the management of a recurrent desmoid tumor of the right flank: a case report

**DOI:** 10.1186/1748-717X-7-178

**Published:** 2012-10-26

**Authors:** Whoon Jong Kil, R Charles Nichols, John W Kilkenny, Soon Y Huh, Meng Wei Ho, Pratibha Gupta, Robert B Marcus, Daniel J Indelicato

**Affiliations:** 1 University of Florida Proton Therapy Institute, Jacksonville, FL, USA; 2 Department of Surgery University of Florida Shands Hospital, Jacksonville, FL, USA; 3 Department of Radiology University of Florida Shands Hospital, Jacksonville, FL, USA

**Keywords:** Proton therapy, Intensity-modulated radiotherapy, Benign tumors, Case report

## Abstract

Desmoid tumors are benign mesenchymal tumors with a strong tendency for local recurrence after surgery. Radiotherapy improves local control following incomplete resection, but nearby organs at risk may limit the dose to the target volume. The patient in this report presented with a recurrent desmoid tumor of the right flank and underwent surgery with microscopically positive margins. Particular problems presented in this case included that the tumor bed was situated in close proximity to the liver and the right kidney and that the right kidney was responsible for 65% of the patient’s renal function. Intensity-modulated radiation therapy plans delivering 54 Gy necessarily exposed the right kidney to a V_18_ of 98% and the liver to a V_30_ of 55%. Proton therapy plans significantly reduced the right kidney V_18_ to 32% and the liver V_30_ to 28%. In light of this, the proton plan was utilized for treatment of this patient. Proton therapy was tolerated without gastrointestinal discomfort or other complaints. Twenty-four months after initiation of proton therapy, the patient is without clinical or radiographic evidence of disease recurrence. In this setting, the improved dose distribution associated with proton therapy allowed for curative treatment of a patient who arguably could not have been safely treated with intensity-modulated radiation therapy or other methods of conventional radiotherapy.

## Background

Desmoid tumor (DT) is a deep-seated fibroblastic neoplasm that arises from musculoaponeurotic stromal tissue. It exhibits slow growth with a strong tendency for local recurrence (LR) and a low metastatic potential. DTs can arise anywhere in the body, but commonly occur in the proximal extremities, trunk, and abdominal wall. Surgery remains the primary treatment for DT with a goal of gross total resection with wide surgical margins. Because of the infiltrative growth pattern of DT, surgical resection alone is associated with a significant LR rate of 24% to 77% [1-3]. Several studies have suggested lower LR rates when adjuvant radiation therapy (RT) is employed [4-6]. Previously, our institution reported improved local-regional control in patients with DT receiving a total radiation dose ≥ 55 Gy after surgical resection [7]. While delivering doses in the range of 55 Gy to extremity lesions can be achieved with 3-dimensioanl conformal RT (3DCRT) or intensity-modulated RT (IMRT) using x-rays - since the targets are generally located away from critical radiosensitive tissues - delivering such doses to truncal targets is more difficult due to the proximity of highly radiosensitive organs, such as the lungs, spinal cord, intestines, liver and kidneys.

Proton therapy (PT) has the potential to improve the therapeutic index in such a setting compared to conventional x-ray-based therapy. While x-rays pass through the patient and leave a track of exposure from the entrance surface to the exit surface of the patient, protons (which are particles with mass) can be accelerated to penetrate into tissue only to the depth of the target. When patients are treated with proton-based radiotherapy most of the radiation energy is discharged at a discrete and predictable depth called the Bragg peak. A "spread-out Bragg peak" can be created to match the exact depth and thickness of the target. The case we present here demonstrates a situation where the improved therapeutic index associated with PT allowed for the potentially curative treatment of a patient who arguably could not have been safely treated with x-rays.

### Case presentation

**Figure 1:**
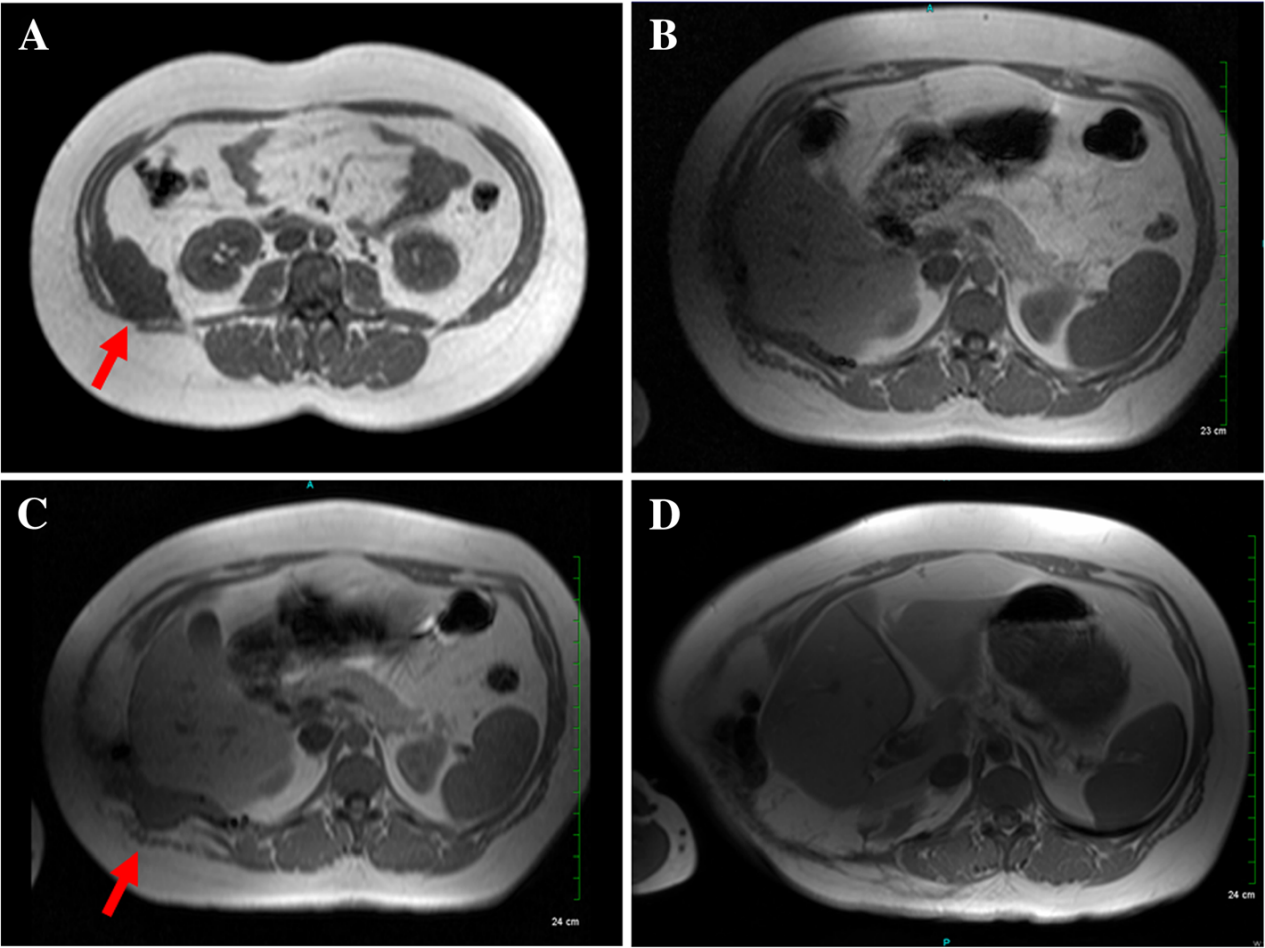
**Serial magnetic resonance imaging (MRI) scans.** (**A**) Taken at the initial diagnosis. The red arrow indicates 7.3 × 4-cm mass on the right posterior-lateral abdominal wall at the level of the flank and right kidney. (**B**) Taken after the initial surgery, showing no evidence of disease. (**C**) Taken 12 months after the initial surgery. The red arrow indicates 6.5 × 2.8-cm recurrent mass on the previous surgical bed. (**D**) Taken at 24 months after completing proton therapy. No evidence of re-recurrence of the tumor.

A 36-year-old white female self-detected a mass in the right flank. Magnetic resonance imaging (MRI) demonstrated a 7.3 × 4-cm lobulated mass along the right posterior-lateral abdominal wall at the level of the right kidney (Figure 1A). Incisional biopsy was performed followed by surgical resection. Final resection margins were microscopically negative. Postoperative MRI of the abdomen demonstrated no residual mass in the right flank (Figure 1B). No adjuvant treatment was recommended.

Approximately 12 months after the surgical resection, the patient noticed a palpable lump in the surgical bed. MRI of the abdomen revealed a 6.5 × 2.8-cm mass involving the right flank with an associated abdominal wall hernia (Figure 1C). Salvage surgery was performed to remove the recurrent mass and repair the hernia. Final histopathology again revealed a benign DT. The microscopic surgical margin was focally positive. The patient’s case was presented at a multidisciplinary tumor board with the recommendation that she receive adjuvant radiotherapy based on the tumor’s recurrent nature and the presence of positive margins.

**Figure 2:**
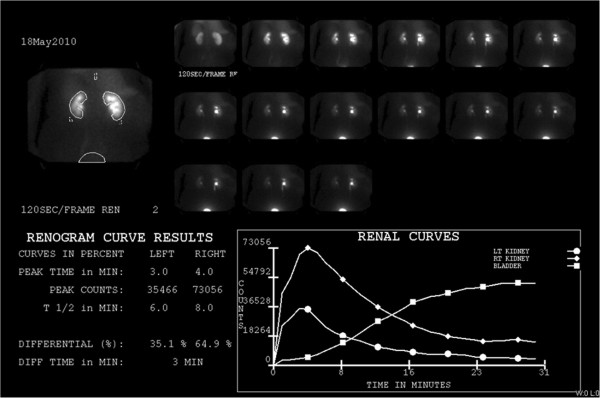
**A nuclear renal scan after intravenous injection of 11.085 mCi of Tc-99m MAG3.** There is decreased flow and uptake within the left kidney compared to the right. Differential renal function is 35% for the left kidney and 65% for the right kidney. There is prompt bilateral excretion with a transit time of three minutes. No evidence of obstruction.

At her initial radiation oncology consultation, the patient demonstrated a long, horizontal incision in the right posterolateral aspect of the abdominal wall that was healing. No suspicious palpable abnormality was appreciated. Given the proximity of the tumor bed to the right kidney, split renal function studies were ordered. Laboratory data showed that blood urea nitrogen and creatinine levels to be within normal ranges (8.0 mg/dL and 0.49 mg/dL, respectively) as well as a glomerular filtration rate of > 60 mL per minute. A nuclear renal scan using Tc-99m MAG3 demonstrated decreased flow and uptake within the left kidney compared to the right kidney (Figure 2). The right kidney was the dominant kidney, comprising 65% of the patient’s renal function. The risks and benefits of adjuvant external-beam RT in this context were discussed with the patient who elected to pursue adjuvant radiotherapy.

**Figure 3:**
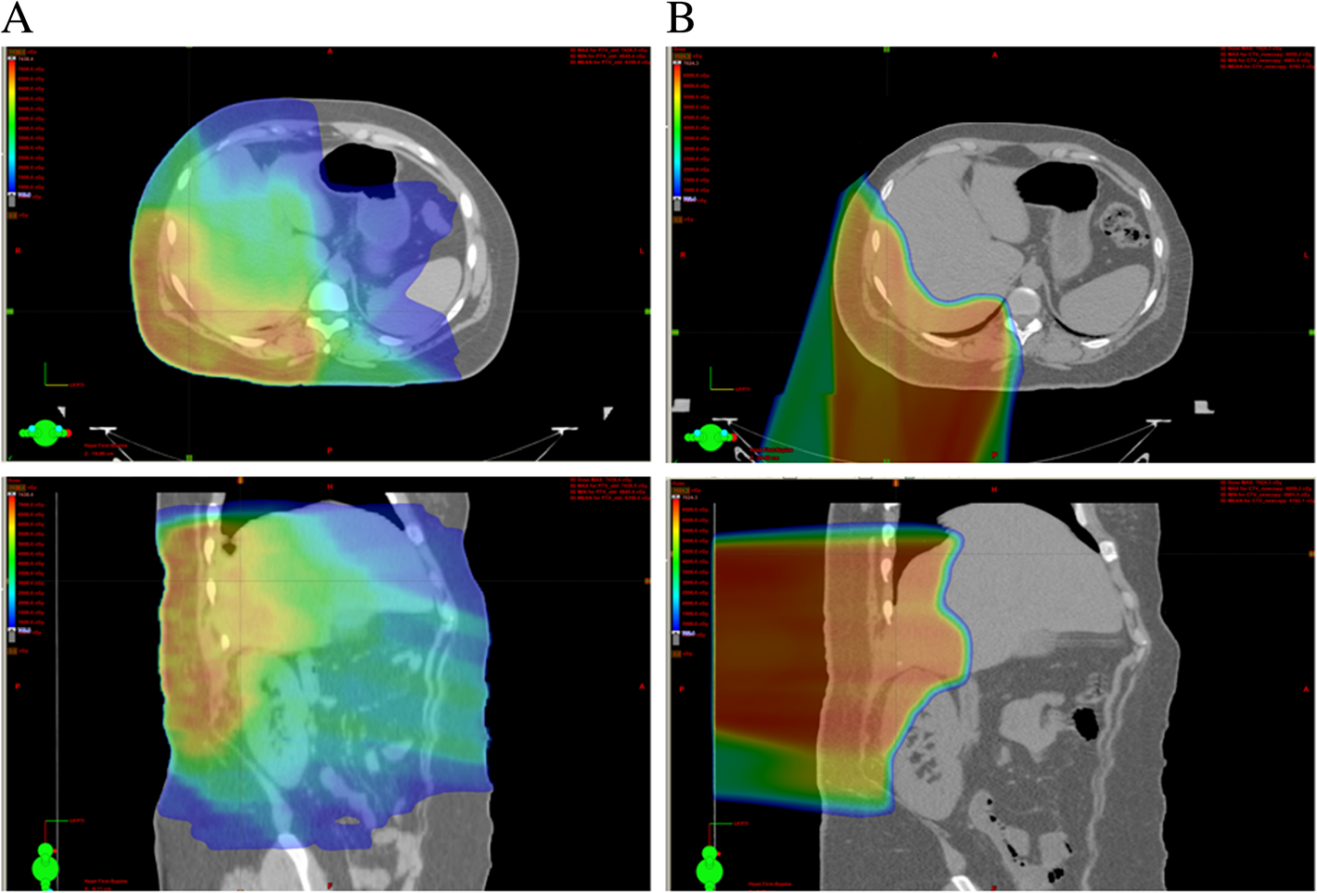
**Colorwash comparisons of the intensity-modulated radiotherapy (IMRT) and proton plans to deliver 59.40 Gy or 59.40 Cobalt Gray Equivalent (CGE) to the planning target volume (PTV).** Dose-volume histograms (DVH) for these plans are shown in Figure 4.

For the RT, an optimized IMRT plan was generated to deliver a dose of 54 Gy (1.8 Gy per fraction) to the tumor bed (Figure 3A). The planning target volume (PTV) consisted of the initial tumor bed plus the recurrent tumor bed with an approximately 6-cm margin on the abdominal wall (but not extending into the abdominal cavity). Ninety-five percent of the PTV volume received 100% of the prescribed target dose and 100% of the PTV volume received 95% of the target dose. Normal-tissue goals of particular interest were as follows: right kidney V_18_ (volume receiving ≥ 18 Gy) to < 70%; left kidney V_18_ to < 30%; liver V_30_ Gy to < 50%; and spinal cord maximum to < 46 Gy. IMRT plans delivering 54 Gy to the PTV, however, necessarily exposed the 98% of the right kidney to ≥ 18 Gy and 55% of the liver to ≥ 30 Gy. Minimizing the right kidney dose was necessarily prioritized because the right kidney was the dominant kidney and responsible for 65% of the patient’s renal function.

To deliver a high dose of radiation to the PTV without compromising the function of the right kidney, PT plans were then generated. The clinical target volume (CTV) and PTV were identical. The proton plan utilized two fields that included posterior-anterior and right posterior-oblique fields. The CTV and PTV coverages were identical. Dose was prescribed in cobalt Gy equivalent (CGE) by use of a relative biological effectiveness of 1.1. The proton plan reduced the right kidney V_18_ to 32% and the liver V_30_ to 28% without adversely affecting other critical organs or compromising target coverage (Figure 3B). Additional benefits from the PT plan compared to the photon IMRT plan included sparing the left kidney from any radiation exposure and lowering the integral dose to the body. In light of these advantages, the PT plan was recommended.

**Figure 4:**
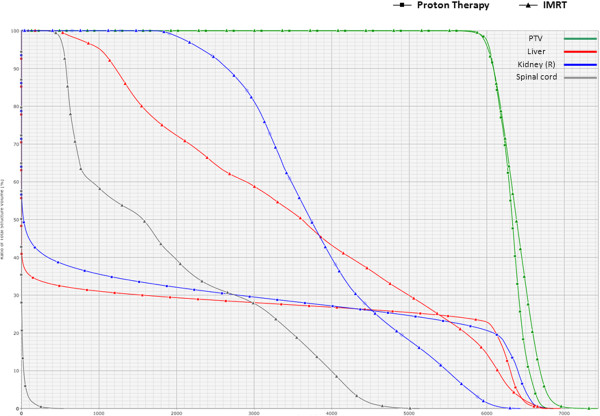
**Dose-volume histogram (DVH) data for the proton plan (delivered) and the corresponding optimized intensity-modulated radiotherapy (IMRT) plan shown in Figure**3. The planning target volume (PTV) dose was 59.40 Gy for the IMRT plan and 59.40 CGE for the proton plan. Normal-tissue exposures for the proton plan were 32% for the right kidney V18 CGE and 28% for the liver V30 CGE 28%. Normal-tissue exposures for the IMRT plan were 98% for the right kidney V18 Gy and 55% for the liver V30 Gy.

**Figure 5:**
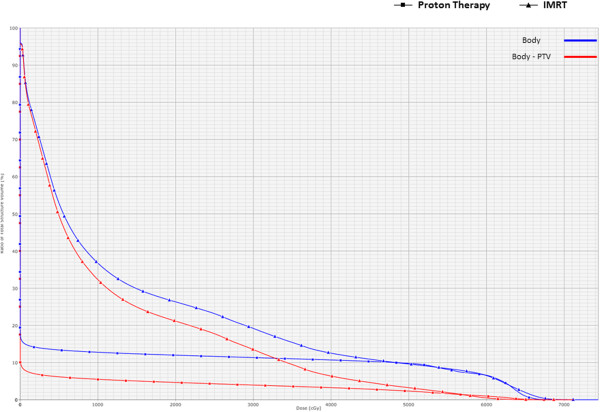
DVH demonstrates reduced total body dose with protons, notably in the low-dose range.

The initial PT plan was to deliver 54 CGE (1.8 CGE per fraction) to the PTV. The patient, however, had a family emergency, resulting in a 2-week treatment delay after delivery of 5.4 CGE. Therefore, the final PTV dose was increased to 59.4 CGE over 33 fractions. Normal-tissue exposures for the PT plan compared to the photon IMRT plan were as follows (with hypothetical optimized IMRT exposures in parentheses): mean liver dose, 17.6 CGE (versus 36.1 Gy); mean right kidney dose, 18.9 CGE (versus 38.8 Gy); mean spinal cord dose, 0.1 CGE (versus 18.9 Gy) (Figure 4). The integral dose to the body was lower with protons, particularly in the low dose range, with greater than a 5-fold reduction in the volume of uninvolved normal tissues receiving a dose of 10 Gy (Figure 5).

The patient tolerated the treatment uneventfully without nausea or other gastrointestinal discomfort. Her only measurable toxicity was grade 1 skin erythema without desquamation in the treated field. Her blood urea nitrogen and creatinine levels were 10.0 mg/dL and 0.7 mg/dL, respectively, with a glomerular filtration rate of greater than 101.2 mL per minute at the completion of PT. Her most recent physical examination 24 months after treatment demonstrates no palpable mass and no skin toxicity. She is without any gastrointestinal toxicity and is working full-time. MRI of the abdomen at 24 months after initiation of PT demonstrates no evidence of recurrent disease (Figure 1D).

## Discussion

DTs are benign tumors with locally aggressive growth and a high rate of recurrence after surgical resection. Adjuvant postoperative RT is regularly utilized at our institution to reduce recurrence risk. A previous study published by the University of Florida evaluating the local-regional control of DTs in an adult cohort showed a 5-year local-regional control rate of 83% [7]. Proton therapy has been demonstrated to reduce gastrointestinal exposure compared to photon-based radiotherapy in the treatment of abdominal malignancies [8,9]. The same principles described in the aforementioned studies allowed for significant normal-tissue sparing in this case. In addition to allowing for the delivery of a radiation dose adequate to secure disease control while avoiding renal and gastrointestinal toxicity, PT also was associated with a significant reduction in total-body radiation exposure compared to the exposure associated with IMRT. Since the correlation between radiation exposure and radiation-induced second malignancies is well established [10-21], and a survival time of 10 years or longer is not uncommon for patients with DTs, reducing the body volume receiving low-dose radiation may be of particular importance in patients for whom a high rate of disease control and long-term survival is expected.

## Conclusion

Proton therepy in this case allowed for the delivery of a radiation dose adequate to achieve local control without exposing the patient to renal or gastrointestinal toxicity. Our pretreatment dosimetry indicated that such a favorable outcome could not have been achieved with IMRT. PT in this case was also associated with a lower integral total body dose than would have been associated with IMRT. The latter finding might be particularly relevant in reducing the risk of late iatrogenic malignancy in a young patient.

## Consent

Written informed consent was obtained from the patient for publication in this case report and any accompanying images. A copy of the written consent is available for review by the Editor-in-Chief of this journal.

## Abbreviations

OARs: Organs at risk; CGE: Cobalt Gray equivalent; CTV: Clinical target volume; DT: Desmoid tumor; Gy: Gray; IMRT: Intensity-modulated radiation therapy; MRI: Magnetic resonance imaging; PT: Proton therapy; RT: Radiation therapy; V_18_: Target or organ volume receiving ≥ 18 Gy; V_30_: Target or organ volume receiving ≥ 30 Gy; V_4_: Target or organ volume receiving ≥ 4 Gy.

## Competing interests

The authors declare that they have no competing interests.

## Authors’ contributions

WJK analyzed the treatments and drafted the manuscript. RCN planned and analyzed the treatments and contributed to the final draft of the manuscript. JWK performed the surgeries and contributed to the final draft of the manuscript. SYH planned the treatments, assisted with analysis, and contributed to the manuscript. MWH planned the treatments, assisted with analysis, and contributed to the manuscript. PG reviewed the imaging and contributed to the manuscript. RBM assisted with planning and analyzing the treatments and contributed to the manuscript. DJI assisted with planning and analyzing the treatments and contributed to the manuscript. All authors read and approved the final manuscript.
